# Effects of Early Intervention with Antibiotics and Maternal Fecal Microbiota on Transcriptomic Profiling Ileal Mucusa in Neonatal Pigs

**DOI:** 10.3390/antibiotics9010035

**Published:** 2020-01-18

**Authors:** Rongying Xu, Jiajia Wan, Chunhui Lin, Yong Su

**Affiliations:** 1 Laboratory of Gastrointestinal Microbiology, Jiangsu Key Laboratory of Gastrointestinal Nutrition and Animal Health, College of Animal Science and Technology, Nanjing Agricultural University, Nanjing 210095, China; 2017105053@njau.edu.cn (R.X.); 2016105047@njau.edu.cn (J.W.); 2016105048@njau.edu.cn (C.L.); 2 National Center for International Research on Animal Gut Nutrition, Nanjing Agricultural University, Nanjing 210095, China

**Keywords:** antibiotic, fecal microbiota transplantation, ileal mucosa, neonatal pig, transcriptomic profiling

## Abstract

This study aimed to investigate the effects of early intervention with antibiotics and maternal fecal microbiota on ileal morphology and barrier function, and transcriptomic profiling in neonatal piglets. Piglets in the amoxicillin (AM), fecal microbiota transplantation (FMT), and control (CO) groups were orally administrated with amoxicillin solution (6.94 mg/mL), maternal fecal microbiota suspension [>10^9^ colony forming unit (CFU)/mL], and physiological saline, respectively. Compared with the CO group, early intervention with AM or FMT significantly decreased ileal crypt depth on day 7 and altered gene expression profiles in ileum on days 7 and 21, and especially promoted the expression of chemokines (*CCL5*, *CXCL9,* and *CXCL11*) involved in the toll-like receptor signaling pathway on day 21. FMT changed major immune activities from B cell immunity on day 7 to T cell immunity on day 21 in the ileum. On the other hand, both AM and FMT predominantly downregulated the gene expression of toll-like receptor 4 (*TLR4*). In summary, both early interventions modulated intestinal barrier function and immune system in the ileum with a low impact on ileal morphology and development.

## 1. Introduction

The early colonization of the gut microbiota, which is considered to be the major antigen challenge for the newborn, is essential for the maturation of the gut-associated lymphoid tissue and for the developmental regulation of the intestinal physiology [1,2,3]. In addition, on molecular levels, members of intestinal microbiota have been reported to have a great capacity to regulate the expression of host genes related to mucosal barrier function and a variety of other intestinal functions, including nutrient absorption, metabolism, angiogenesis, and intestinal maturation [4]. During early life stages, intestinal microbiota is dynamic and can be easily influenced by environmental conditions [5,6,7]; therefore, modulating the intestinal microbiota development in early life as a strategy to maintain host health in later life has drawn wide attention.

Antibiotics can cause changes in the normal development of intestinal microbiota, generally coinciding with a decrease in phylogenetic diversity [8,9,10]. Amoxicillin, a frequently used antibiotic, produced simplified hindgut bacteria communities with decreased counts in mice [11], and reduced the numbers of gut microbiota diversity in rats [12]. Amoxicillin treatment fundamentally eliminates *Lactobacillus* spp. in the small intestine [13] and these microbiota changes reduce the expression of major histocompatibility complex (MHC) class I and II genes in the small and large intestine in suckling rats [14]. However, the impact of early intervention with antibiotics on intestinal function in neonatal pigs is not fully clear.

Another modulation strategy is fecal microbiota transplantation (FMT), which can normalize the composition and functionality of gut microbiota [15]. It refers to the process of transplanting the functional flora of donor feces into the gastrointestinal tract of the recipient and reconstructing new intestinal microbiota, which is mainly used in humans [16]. Early FMT treatment not only induced changes in offspring’s gut microbiota composition (mostly in the ileum), but also altered the abundances of predicted bacterial pathways, affected intestinal morphology, and modulated duodenal gene expression in newborn pigs [17]. Taken together, FMT demonstrates an extensive impact on early-life intestinal microbiota and host phenotype is changed accordingly. Maternal fecal microbiota, as an environmental factor, inevitably comes into contact with neonate at birth. Our previous study suggested maternal fecal microbiota may play an important role in the process of gut microbiota colonization in piglets [18]; thus, this early intervention may further impact the intestinal development and function of neonatal pigs.

In the present study, oral administration with amoxicillin or maternal fecal microbiota was performed in an early intervention model on pig gut microbiota. Although our previous study investigated short- and long-term effects of early intervention with amoxicillin and maternal fecal microbiota on intestinal microbiota and metabolites in newborn piglets [19], the corresponding impact on intestinal function is yet unclear. Therefore, the aim of this study was to investigate the effect of early oral administration of amoxicillin and maternal fecal microbiota transplantation on the ileal mucosa gene expression and intestinal function in neonatal piglets.

## 2. Materials and Methods

### 2.1. Ethics Statement

The present study followed the guidelines for animal care and use of Nanjing Agricultural University (Nanjing, Jiangsu province, China) and the whole experiment process was under the support of the Animal Care and Use Committee (SYXK2017-0027).

### 2.2. Donor Material Preparation

The preparation of maternal fecal microbiota suspension was adapted from a previous study [20]. Briefly, mixing fresh fecal samples from candidate pregnant sows with anaerobic sterile 0.9% NaCl solution (1:5) and sterile filtered. The obtained filtrate was centrifuged (2000 rpm, 10 min) and then the supernatant was dispensed to 10 ml sterile tubes and frozen at −80 °C. The entire preparation process was under anaerobic condition.

### 2.3. Animal Experiment and Sampling

Five litters of healthy neonatal 0-day-old piglets (Duroc × Landrace × Yorkshire, nine piglets in each litter) were used in this study. Each litter was randomly allocated into the CO, AM, or FMT groups, with three piglets in each group. On days 1–6, piglets in the maternal fecal microbiota transplantation (FMT) group were orally administered with 3 ml fecal microbiota suspension [>10^9^ colony forming unit (CFU)/mL] at 8:00 am every day, piglets in the amoxicillin treatment (AM) group and the control (CO) group were orally supplemented with the same volume of amoxicillin (6.94 mg/mL) or physiological saline (0.9% NaCl), respectively. All piglets had access to breast milk and water ad libitum and had no other creep feed throughout the experiment period.

At 8:00 am of days 7 and 21 (weaning day), one piglet per group in each litter was randomly selected and then anesthetized and euthanized with a jugular vein injection of 4% sodium pentobarbital solution (40 mg/kg body weight). Blood samples were taken from the anterior vena cava and centrifuged at 3000 rpm for 15 min, the serum was then stored at −28 °C for the analysis of cytokine concentrations. Segments of the distal ileum were removed and fixed by immersion in 10% (v:v) phosphate buffered formalin for histologic study. The ileal mucosa was collected by scraping with sterile glass microscope slides and rapidly stored at −80 °C for further analysis.

The gut microbiota of piglets used in this study have been analyzed in our previous study [19].

### 2.4. Morphometric Analysis

Ileum tissue samples for the morphometric study were dehydrated and embedded in paraffin wax, sectioned at 4 μm, and stained with hematoxylin and eosin. Villus height and crypt depth were measured by a NIS-Elements F software in a bright field microscope (Nikon, Japan). Villus height was measured from the top of the villus to the top of the lamina propria. Crypt depth was measured from the base upwards to the region of transition between the crypt and villus.

### 2.5. Serum Cytokines Analysis

The concentrations of interferon gamma (IFN-γ), interleukin 1 alpha (IL-1α), interleukin 1 beta (IL-1β), interleukin 2 (IL-2), interleukin 4 (IL-4), interleukin 6 (IL-6), interleukin 8 (IL-8), interleukin 10 (IL-10), interleukin 12 (IL-12), and tumor necrosis factor alpha (TNF-α) in the serum were determined with the ELISA kit (the US China Business Association (USCN), USA), according to the manufacturer’s instructions.

### 2.6. RNA Isolation and Sequencing

The RNA was isolated from ileum mucosa with RNAiso Plus Total RNA extraction reagent (Takara) according to the instructions, and the integrity was inspected through an Agilent Bioanalyzer 2100 (Agilent technologies, Santa Clara, CA, USA). Three samples in each group were randomly selected for sequencing for reducing the cost of the experiment. The rRNA of the purified RNA was removed with rRNA removal bead, and then the RNA was divided into fragment and converted to cDNA. The cDNA was purified, and sequencing adaptors were attached to the fragments. Suitable fragments were isolated and amplified by PCR. The libraries outcomes were sequenced by Illumina HiSeq 2500. The average number of reads of samples was 8.54G, and the Q20 ratios were all higher than 90%. Qualified reads were further analyzed. The information of quality control during sequencing is showed in Supplementary Table S1. The preparation and sequencing of libraries was performed by Shanghai Biotechnology Corporation (Shanghai, China).

### 2.7. Quantitative Real-Time PCR

Quantitative analysis real-time PCR was performed on ABI 7300 (Applied Biosystems, Foster City, CA, USA) using SybrGreen (Roche, Switzerland) according to the manufacturer’s instructions. The expression of inflammatory cytokines genes, tight junction genes, and intestinal development genes were determined. Besides, the gene expression of *C-C motif chemokine ligand 4* (*CCL4*) [21], *C-C motif chemokine ligand 5* (*CCL5*) [22], *C-X-C motif chemokine ligand 9* (*CXCL9*) [23], *CD19 molecule* (*CD19*) [24], *inducible T cell costimulator* (*ICOS*) [25], and *C-X-C motif chemokine receptor 6* (*CXCR6*) [26] was determined to validate the result of RNA-sequencing (RNA-seq). The primers used are shown in Supplementary Table S2. The expression of the genes was calculated by normalizing the results to the expression of *β-actin* gene with formula 2^−ΔΔCt^.

### 2.8. Data Analysis 

The data analysis of morphology, quantitative real-time PCR, and serum cytokines was implemented with SPSS (version 20) as a randomized block design. A litter was regarded as an experimental unit, meaning *n* = 5. The differences among groups were evaluated using one-way analysis of variance (ANOVA) with *p* < 0.05 as the criteria to declare significantly different.

Regarding the RNA-seq, the raw data were filtered with Seqtk, and then the preprocessed data underwent genome mapping by spliced mapping algorithm of Hisat2 (version: 2.0.4) [27]. After comparison, the fragment numbers of each gene were counted with Stringtie (version: 1.3.0) [28,29] and then were normalized by TMM (trimmed mean of M values) [30]. Finally, the fragments per kilobase of exon model per million mapped reads (FPKM) of each gene were calculated using the Perl script. The edgeR [31] was used to analyze the differentially expressed genes among samples and the fold-change (FC) values were calculated based on FPKM. The genes with *p* value < 0.05 and FC > 2 or < 0.5 are thought to be significant differences and are selected for further analysis.

The gene ontology (GO) enrichment analysis and Kyoto Encyclopedia of Genes and Genomes (KEGG) Pathway enrichment analysis was performed on the Database for Annotation, Visualization and Integrated Discovery (DAVID) Bioinformatics Resources 6.8 (https://david.ncifcrf.gov/tools.jsp, accessed 15 June 2018). The Fisher’s exact test was selected as the statistical method, and false discovery rate (FDR) correct method was the FDR value. The difference was seen as significant when *p* < 0.05.

## 3. Results

### 3.1. Ileal Morphological Structure

As shown in Table 1, the crypt depth of the ileum both in AM and FMT groups were significantly lower than that in the CO group on day 7 (*p* < 0.05). However, no significant difference in ileal morphology was observed in all groups on day 21.

### 3.2. Serum Inflammatory Cytokines Concentrations

On day 7, there were no changes in concentrations of all detected cytokines among groups. On day 21, FMT significantly increased the concentration of IL-8 (*p* < 0.05) in plasma compared to the CO group. The concentration of IFN-γ was significantly declined (*p* < 0.05) in the AM compared to the FMT and CO. No significant difference in the concentration of IL-2, IL-6, IL-10, IL-12, and TNFα was observed among three groups (Table 2).

### 3.3. Expression of Genes Related to Intestinal Epithelial Tight Junction Proteins, Toll-Like Receptors, and Inflammatory Cytokines in the Ileum

**Figure 1:**
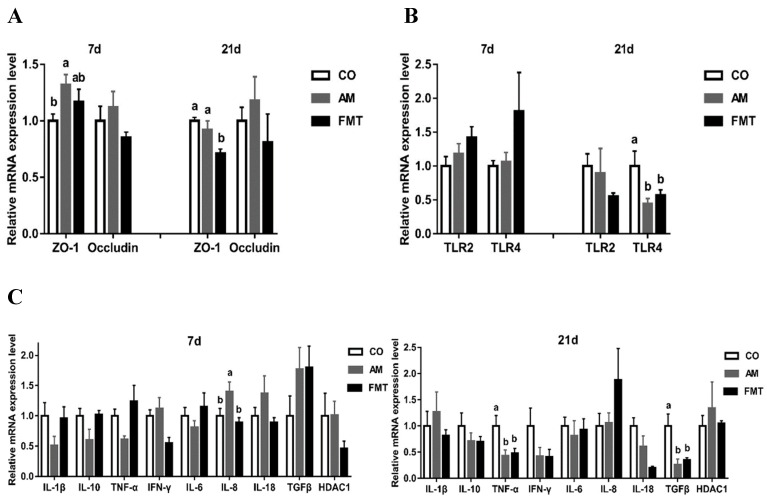
Relative mRNA expression of genes related to intestinal epithelial tight junction proteins (**A**), toll-like receptors and (**B**) inflammatory cytokines (**C**) in the ileum in the amoxicillin (AM), fecal microbiota transplantation (FMT), and control (CO) groups on days 7 and 21. Columns with different letters differ significantly (*p* < 0.05).

Compared with the CO group, the mRNA expression of *ZO-1* was higher (*p* < 0.05) in the AM group on day 7, while was lower (*p* < 0.05) in the FMT group on day 21. No significant difference in the mRNA expression of occludin was observed among the three groups (Figure 1A). On day 21, the FMT and AM group showed a significant reduction (*p* < 0.05) of toll-like receptor 4 (*TLR4*) mRNA expression compared to the CO group. No significant difference in the mRNA expression of toll-like receptor 2 (*TLR2*) was observed among the three groups (Figure 1B). On day 7, the mRNA expression of pro-inflammatory cytokine *IL-8* in the ileum in the AM group was significantly increased (*p* < 0.05) compared to the CO group. On day 21, for pro-inflammatory cytokine *TNF-α* and anti-inflammatory cytokine *TGF-β*, the mRNA expression in the FMT and AM group was significantly lower (*p* < 0.05) than that of the CO group. No significant difference in the mRNA expression of *IL-1β*, *IL-10*, interferon gamma (*IFN-γ*), *IL-6*, *IL-18*, and histone deacetylase 1 (*HDAC1*) was observed among the three groups (Figure 1C).

### 3.4. Expression of Genes Related to Intestinal Development in the Ileum

No significant difference was observed in the mRNA expression of the intestinal development-related genes including insulin-like growth factor 1 receptor (*IGF-1R*), insulin-like growth factor 1 (*IGF-1*), glucagon-like peptide 2 (*GLP-2*), and epidermal growth factor (*EGF*) in the ileum among three groups (Supplementary Figure S1).

### 3.5. Transcriptomic Profiling of the Ileal Mucosa

**Figure 2:**
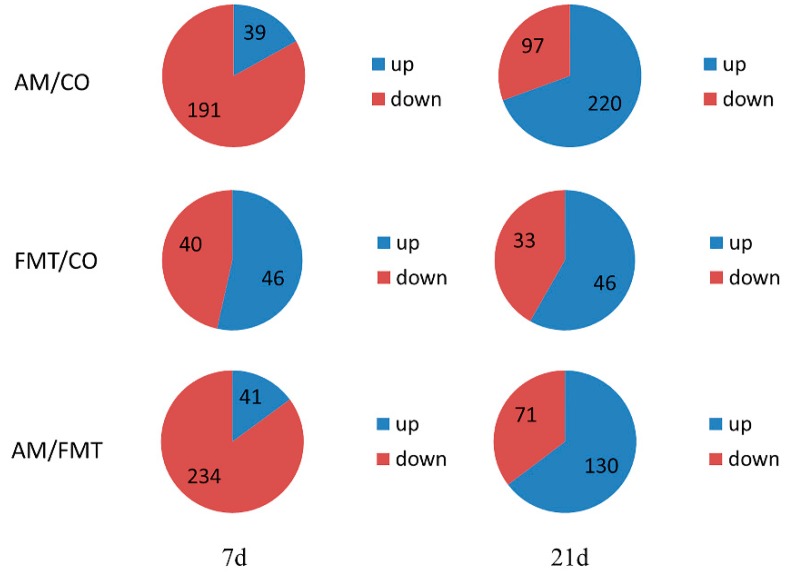
Numbers of the total differentially expressed genes as well as the up- and down-regulated genes in the ileum mucosa of pigs among the amoxicillin (AM), fecal microbiota transplantation (FMT), and control (CO) groups on days 7 and 21.

On day 7, there were 230 differentially expressed genes between AM and CO groups, 86 between FMT and CO, and 275 between AM and FMT, respectively. On day 21, there were 317 differentially expressed genes between AM and CO groups, 79 between FMT and CO groups, and 201 between AM and FMT (Figure 2). 

**Figure 3:**
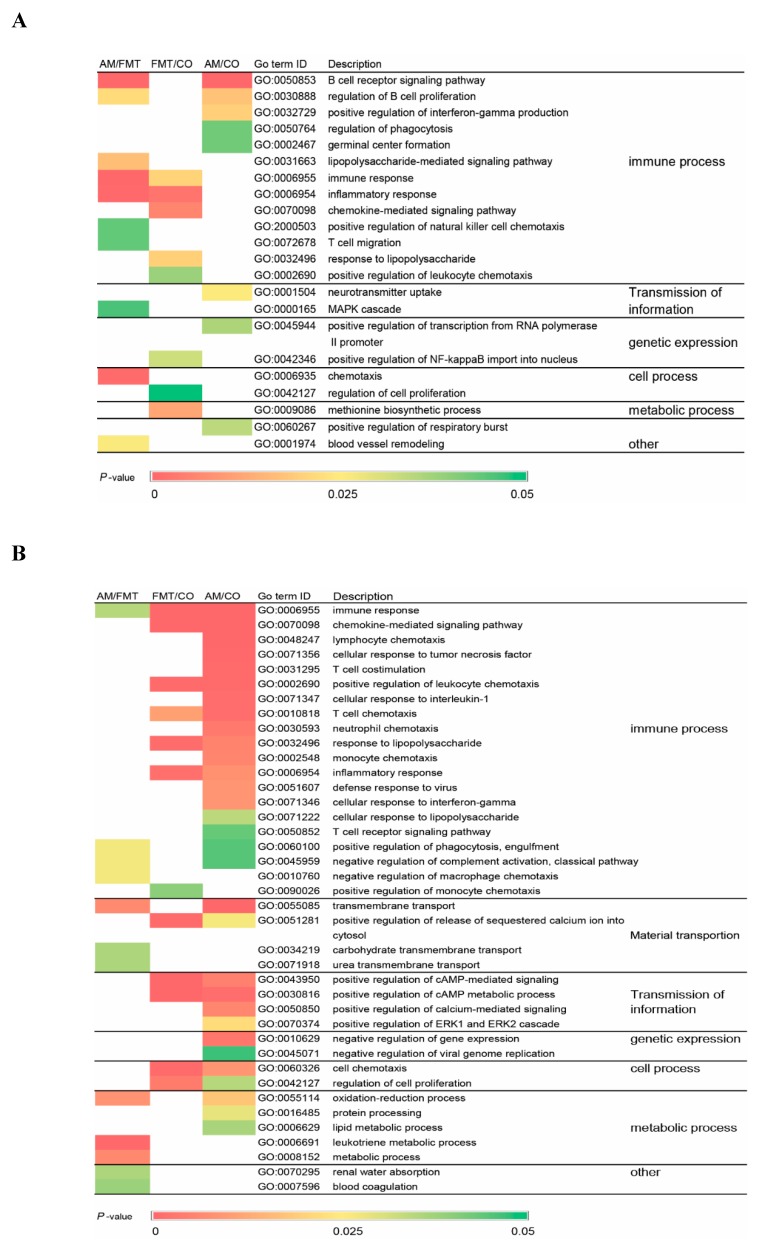
Gene ontology (GO) term enrichment analysis of the biological processes of the differentially expressed genes in the ileum mucosa of pigs induced by the amoxicillin (AM), fecal microbiota transplantation (FMT), and control (CO) groups on (**A**) days 7 and (**B**) 21.

As show in Figure 3, gene ontology (GO) enrichment analysis were performed. On day 7, a total of 22 terms were significantly affected, and most of them were related to immunity (Figure 3A). On day 21, 39 terms were significantly altered, and most of them were related to the immunity process, material transportation, and transmission of information. Among them, 31 terms were significantly affected by AM (Figure 3B). FMT altered major immune activities from B cell immunity on day 7 to T cell immunity on day 21. Supplementary Table S3 shows the up- or down-regulated GO terms in comparisons among groups. On day 7, GO terms about B cell receptor signaling pathway, regulation of B cell proliferation, and positive regulation of interferon-gamma production were significantly downregulated in AM/CO and AM/FMT, and immune response, inflammatory response, and chemokine-mediated signaling pathway were significantly upregulated in FMT/CO. On day 21, compared with the control group, AM significantly upregulated immune response, chemokine-mediated signaling pathway, and lymphocyte chemotaxis, and FMT upregulated positive regulation of cAMP metabolic process, positive regulation of cAMP-mediated signaling, and chemokine-mediated signaling pathway. In AM/FMT, negative regulation of complement activation, classical pathway, leukotriene metabolic process, and transmembrane transport were significantly upregulated, but carbohydrate transmembrane transport was significantly downregulated (Supplementary Table S3).

**Figure 4:**
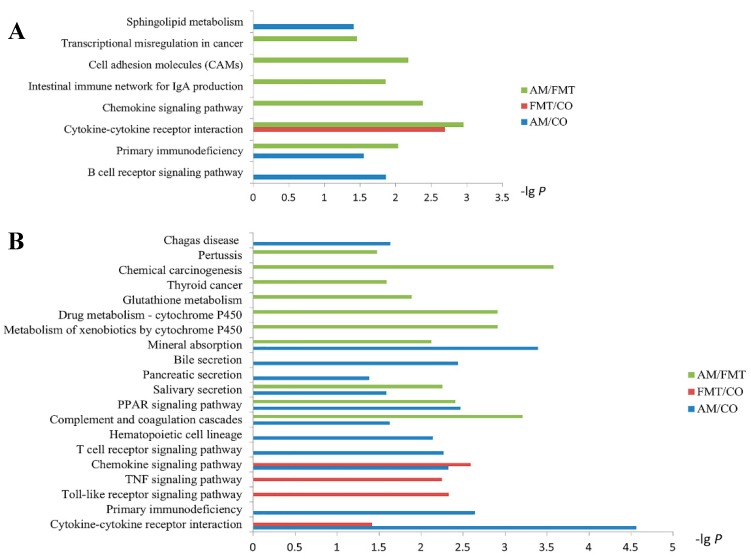
Kyoto Encyclopedia of Genes and Genomes (KEGG) pathway analysis of the differentially expressed genes in the ileum mucosa among the amoxicillin (AM), fecal microbiota transplantation (FMT), and control (CO) groups on (**A**) days 7 and (**B**) 21.

KEGG pathway enrichment is displayed in Figure 4. On day 7, the cytokine–cytokine receptor interaction was significantly affected by FMT. Sphingolipid metabolism, primary immunodeficiency, and the B cell receptor signaling pathway were significantly affected by AM. Compared with FMT, cell adhesion molecules (CAMs), the intestinal immune network for IgA production, the chemokine signaling pathway, primary immunodeficiency, and the cytokine–cytokine receptor interaction were significantly affected by AM (Figure 4A).

On day 21, the cytokine–cytokine receptor interaction and chemokine signaling pathway were significantly affected by AM and FMT. Primary immunodeficiency, the T cell receptor signaling pathway, hematopoietic cell lineage, complement and coagulation cascades, the PPAR signaling pathway, salivary secretion, pancreatic secretion, bile secretion, and mineral absorption were significantly affected by AM. The toll-like receptor signaling pathway, TNF signaling pathway, and graft-versus-host disease (not shown) were significantly affected by FMT. Compared with FMT, glutathione metabolism, metabolism of xenobiotics by cytochrome P450, drug metabolism—cytochrome P450, mineral absorption, salivary secretion, the PPAR signaling pathway, and complement and coagulation cascades were significantly affected by AM (Figure 4B).

### 3.6. Validation of RNA-Sequencing by Quantitative Real-Time PCR

In order to validate the reliability of RNA-seq results, we validated six genes including *CCL4*, *CCL5*, *CXCL9*, *CD19*, *ICOS,* and *CXCR6* by quantitative real-time PCR (Supplementary Figure S2). The results showed that the expressions of validated genes were consistent with the results of RNA-seq.

## 4. Discussion

In a previous study, we reported the effects of early intervention with amoxicillin and maternal fecal microbiota on gut microbiota and metabolite profiles in neonatal piglets [19]. Here, we investigated the effects of these two early interventions on intestinal function in the ileum of neonatal piglets by transcriptome analysis, quantitative real-time PCR analysis, as well as intestinal morphology and serum cytokines analysis. The gene expression landscapes should be a valuable resource for researchers interested in exploring molecular mechanisms for the effects of antibiotic administration on neonatal pig ileum. 

### 4.1. Effects of Early Maternal Fecal Microbiota and Antibiotics Intervention on Intestinal Morphology and Barrier Function in the Ileum

The morphology of the intestinal mucosa can reveal some information about gut health. A decrease in crypt depth suggests a reduction in tissue turnover and the surface area for nutrient absorption [32]. In the present study, both amoxicillin and maternal fecal microbiota supplementation decreased crypt depth in the ileum on day 7, which indicated a negative effect on intestinal structure, whereas there was no significant difference on day 21 among the three groups. Similarly, it was reported that the supplementation of antibiotics could decrease colonic crypt depth [33]. In a recent study, treatments of fecal microbiota transplantation from different breeds of swine all exerted no effect on ileal crypt depth in suckling pigs in the long term [34]. Combined with mRNA expression of genes related to intestinal development, these two interventions almost had no effect on ileal development both on days 7 and 21. Thus, in this study, we consider that early intervention with antibiotic or maternal fecal microbiota has a minimal influence on ileal structure on days 7 and 21. 

A complete intestinal barrier can isolate the entry of enteric toxic macromolecules and harmful bacteria and is essential for protecting the gut. The tight junction on the intestinal mucosa is an important part of the intestinal barrier, which prevents the spread of bacteria and other antigens in the epithelium [35]. ZO-1 and occludin, two important tight junction proteins, are commonly used to measure intestinal barrier and permeability [36]. The AM upregulated the mRNA expression of *ZO-1* in the ileum on day 7 in piglets, suggesting that the administration of antibiotics may affect intestinal barrier function and the integrity of the intestinal mucosal structure by *ZO-1*. Toll-like receptors (TLRs) were identified to recognize pathogen ligands and regulate the immune response of the intestinal epithelium by transmitting intestinal bacterial signals [37]. TLR4 is an important transmembrane receptor of LPS, which is responsible for LPS-induced inflammatory cytokines production. On day 21, the decline in *TLR4* mRNA expression in the AM group suggested that antibiotics may affect the LPS/TLR4 signal transduction pathway [38]. 

### 4.2. Effects of Early Maternal Fecal Microbiota and Antibiotics Intervention on Transcriptomic Profiles in the Ileum

Generally speaking, the total numbers of DEGs between day 7 and day 21 are roughly equal in three comparisons (AM/CO, FMT/CO, AM/FMT), but actually, the proportion of upregulated and downregulated genes were significantly altered in AM/CO and AM/FMT. Then, GO and KEGG analyses were performed on DEGs in each comparison. The data showed that, in these comparisons, the number of GO terms and enriched KEGG pathways in a pattern of time effect were greater on day 21 than day 7.

Microbiota colonization after birth is the most important trigger for immune system development [39] and early modulation on microbiota is bound to modify the future immune phenotype of the host [2]. Ileum is considered as the primary site where the gut mucosal immunity is generated, and that is why we focus on its transcriptomic profile. In our previous study, amoxicillin significantly decreased the relative abundance of pathogenic bacteria *Streptococcus* and *Proteobacteria* compared to the control group respectively, on day 7 and 21 in neonatal piglet ileums [19]. *Proteobacteria* is a microbial signature of dysbiosis in gut microbiota [40]. In our results, almost half of the GO terms were involved in immune functions in the ileum whether on day 7 or 21. Especially in AM/CO, compared with day 7 (GO terms mainly related to B cell immunity, which were downregulated in Supplementary Table S3 ), more GO terms of the immune process, mainly including T cell-mediated immune responses and chemotaxis of immunocytes, were altered predominantly in the ileum on day 21. Compared with the CO group, AM significantly downregulated GO terms B cell receptor signaling pathway, regulation of B cell proliferation, and positive regulation of interferon-gamma production on day 7, but immune response, chemokine-mediated signaling pathway, and lymphocyte chemotaxis were upregulated (Supplementary Table S3). It has been reported that antibiotic administration could alter the gene expression profile of the small intestine and increase expression of genes involved in immune functions in the ileum [41], which were consistent with our results. In FMT/CO, also compared with day 7, GO terms related to the immune process did not vary greatly in the ileum on day 21, primarily involving inflammatory response and chemotaxis of immunocytes. It also has been reported that early intervention with fecal microbiota transplantation could permanently change levels of systemic regulatory T cells and cytokine production [2]. 

According to results of KEGG pathway analysis, we observed that the major significant pathways were related to chemokine-mediated pathways both in AM/CO and FMT/CO on day 21, and interestingly, almost all expression of chemokine DEGs were upregulated, such as *C-C motif chemokine ligand 5* (*CCL5*), *C-X-C motif chemokine ligand 9* (*CXCL9*), and *C-X-C motif chemokine ligand 11* (*CXCL11*). Furthermore, we looked into the DEGs (including *CCL4*, *CCL5, CXCL9,* and *CXCL11*) enriched in the toll-like receptor signaling pathway in AM/CO and FMT/CO on day 21 (Table 3), and tried to interpret the effects of DEGs on intestinal function. Studies have shown that the upregulated genes (*CCL4*, *CCL5*) enhanced the chemotaxis of NK cells [42], and *CXCL9* and *CXCL11* strengthened T cell chemotaxis [43]. Consequently, compared with the CO group, both AM and FMT groups enhanced the expression of inflammatory factors in the ileum on day 21. But it was unclear whether these impacts were good or bad for the host health according to the data we had so far. T cells play an important role in the autoimmune response, and *ZAP70* is a critical cytoplasmic tyrosine kinase involved in multiple T-cell receptor signaling pathways [44,45]. In AM/CO, the mRNA expression of *ZAP70*, *CD8B*, *CD3G,* and *CD3D* were all upregulated in the T cell receptor signaling pathway (Table 3), indicating the activation of the T cell [46].

## 5. Conclusions

This study investigated the effects of two early microbial interventions on intestinal function in ileum in neonatal piglets. The results indicated that early intervention with antibiotics or maternal fecal microbiota significantly altered gene expression profiles in ileum on days 7 and 21, and notably promoted the expression of chemokines involved in the toll-like receptor signaling pathway on day 21. Moreover, these early interventions to some extent changed intestinal barrier function, although there was a low impact on ileal morphology and development. The data of this study could be a valuable reference for the further research on exploring molecular mechanisms for the effects of antibiotic administration on neonatal pig ileum.

## Figures and Tables

**Table 1:** Morphology analysis of ileum in neonatal pigs in the amoxicillin (AM), fecal microbiota transplantation (FMT), and control (CO) groups on days 7 and 21.

Item	7 d			21 d		
	CO	AM	FMT	CO	AM	FMT
Villus height, μm	694.65 (66.66)	679.60 (26.00)	607.97 (48.76)	264.69 (3.57)	271.55 (8.48)	222.02 (44.56)
Crypt depth, μm	73.45 (1.67) ^a^	63.14 (4.38) ^b^	56.92 (0.91) ^b^	73.34 (1.18)	67.39 (1.01)	72.62 (3.81)
Villus height/Crypt depth	9.47 (0.95)	10.90 (0.57)	10.67 (0.77)	3.61 (0.06)	4.03 (0.13)	3.85 (0.53)

Data were presented as mean (SEM), *n* = 5. Mean values within a line with different superscript letters differ significantly (*p* < 0.05). SEM: standard error of mean.

**Table 2:** Concentration of serum cytokines of piglets in the amoxicillin (AM), fecal microbiota transplantation (FMT), and control (CO) groups on days 7 and 21.

Items, ng/mL	7 d			21 d		
	AM	FMT	CO	AM	FMT	CO
IFN-γ	16.12 (1.17)	17.65 (0.77)	17.61 (1.35)	13.26 (0.89) ^b^	19.35 (1.11) ^a^	17.66 (1.55) ^a^
IL-2	1.27 (0.25)	1.05 (0.12)	1.35 (0.19)	0.86 (0.14)	0.80 (0.07)	0.92 (0.1)
IL-6	0.820 (0.14)	0.67 (0.12)	0.91 (0.1)	0.25 (0.051)	0.19 (0.04)	0.26 (0.05)
IL-8	0.26 (0.03)	0.19 (0.03)	0.18 (0.03)	0.41 (0.05) ^ab^	0.47 (0.04) ^a^	0.34 (0.03) ^b^
IL-10	2.39 (0.27)	2.00 (0.28)	2.78 (0.32)	0.82 (0.12)	0.64 (0.12)	0.95 (0.15)
IL-12	0.46 (0.09)	0.44 (0.06)	0.55 (0.10)	0.77 (0.04)	0.74 (0.07)	0.76 (0.07)

Data were presented as mean (SEM), *n* = 5. Mean values within a line with different superscript letters differ significantly (*p* < 0.05). SEM: standard error of mean. IFN-γ: interferon gamma; IL-2: interleukin 2; IL-6: interleukin 6; IL-8: interleukin 8; IL-10: interleukin 10; IL-12: interleukin 12.

**Table 3:** Expression of genes related to the toll-like receptor signaling pathway and the T cell receptor signaling pathway in the ileum mucosa of piglets in the amoxicillin (AM), fecal microbiota transplantation (FMT), and control (CO) groups on days 7 and 21.

Gene	7 d			21 d		
	AM/CO	FMT/CO	AM/FMT	AM/CO	FMT/CO	AM/FMT
*CCL4*	NO	NO	DOWN	UP	NO	NO
*CCL5*	NO	NO	NO	UP	UP	NO
*CXCL9*	NO	UP	NO	UP	UP	NO
*CXCL11*	NO	NO	NO	UP	UP	NO
*ZAP70*	NO	NO	NO	UP	NO	NO
*CD8B*	NO	NO	NO	UP	NO	NO
*CD3G*	NO	NO	NO	UP	UP	NO
*CD3D*	NO	NO	NO	UP	NO	NO

UP: upregulated; DOWN: downregulated; NO: no significant change or 0.5 < fold change < 2; *CCL4*: C-C motif chemokine ligand 4; *CCL5*: C-C motif chemokine ligand 5; *CXCL9*: C-X-C motif chemokine ligand 9; *CXCL11*: C-X-C motif chemokine ligand 11; *ZAP70*: tyrosine-protein kinase ZAP-70; *CD8B*: CD8b molecule; *CD3G*: CD3g molecule; *CD3D*: CD3d molecule.

## Supplementary Materials

The following are available online at https://www.mdpi.com/2079-6382/9/1/35/s1, Figure S1: Effects of early intervention with maternal fecal microbiota and antibiotics on intestinal development gene of the ileum mucosa among the amoxicillin (AM), fecal microbiota transplantation (FMT) and control (CO) groups on days 7 and 21, Figure S2: The qPCR validation of the RNA-seq. The results are displayed as the values of fold changes in the CO group on days 7 and 21, and the qPCR data are presented as the means ± SEM (n = 5). Values with different lowercase letter superscripts indicate a significant difference (*p* < 0.05), and those with the same letter superscripts indicate no significant difference (*p* > 0.05). CO: control; AM: amoxicillin; FMT: fecal microbiota transplantation. *CCL4*: C-C motif chemokine ligand 4; *CCL5*: C-C motif chemokine ligand 5; *CD19*: CD19 molecule; *CXCL9*: C-X-C motif chemokine ligand 9; *CXCR6*: C-X-C motif chemokine receptor 6; *ICOS*: inducible T cell costimulator. Table S1: RNA and sequencing data quality control results, Table S2: Primers lists used for real-time PCR assay in this study, Table S3: The regulated gene ontology (GO) terms in the ileum mucosa of piglets in the amoxicillin (AM), fecal microbiota transplantation (FMT) and control (CO) groups on days 7 and 21.
