# Supplementary material for: Effects of Early Intervention with Antibiotics and Maternal Fecal Microbiota on Transcriptomic Profiling Ileal Mucusa in Neonatal Pigs
Source: Antibiotics (Basel). 2020 Jan 18;9(1):35. doi: 10.3390/antibiotics9010035 (PMC7168243; doi:10.3390/antibiotics9010035)
Supplement: Supplementary file 1 [file antibiotics-09-00035-s001.pdf]

Table S1. RNA and sequencing data quality control results.

Sample [3]	RIN [1]	28S/18S	Orientation	Raw reads	Clean reads	Q20 [2] Value
A-7-1	9.9	2.2	Forward/Reverse	52,626,650	49,493,099	93.79
A-7-2	9.4	1.8	Forward/Reverse	65,172,256	61,339,453	93.85
A-7-3	9.4	1.6	Forward/Reverse	52,184,392	48,308,794	92.53
A-21-1	9.8	2.2	Forward/Reverse	65,375,508	61,737,091	93.69
A-21-2	10.0	2.2	Forward/Reverse	63,130,954	58,822,034	92.76
A-21-3	9.9	2.1	Forward/Reverse	52,378,470	49,102,134	93.30
S-7-1	9.8	2.0	Forward/Reverse	51,769,926	48,551,144	93.68
S-7-2	9.7	2.6	Forward/Reverse	51,973,884	48,915,040	93.93
S-7-3	9.9	2.3	Forward/Reverse	56,478,666	53,034,760	93.55
S-21-1	9.9	2.4	Forward/Reverse	50,999,520	47,905,709	93.60
S-21-2	9.9	1.9	Forward/Reverse	65,885,658	62,039,264	93.48
S-21-3	9.6	1.8	Forward/Reverse	52,273,302	47,750,614	91.71
F-7-1	10.0	2.3	Forward/Reverse	47,341,580	44,635,381	94.23
F-7-2	9.3	2.3	Forward/Reverse	56,505,676	53,306,031	93.81
F-7-3	9.8	2.3	Forward/Reverse	65,404,146	61,675,167	93.67
F-21-1	9.5	2.4	Forward/Reverse	68,286,012	64,035,243	93.30
F-21-2	9.4	2.3	Forward/Reverse	72,320,210	68,540,614	94.12
F-21-3	9.9	2.4	Forward/Reverse	33,871,056	32,279,512	94.79

[1] RIN = RNA Integrity Number

[2] Q20 = bases of Q≥20 / all bases of sequencing

[3] A = amoxicillin; S = control; F = fecal microbiota transplantation; 7 = day 7; 21 = day 21.

Table S2. Primers lists used for real-time PCR assay in this study [1].

Gene	Sequence 5'–3'
CCL4	F: CATGAAGCTCTGCGTGACTG
	R: ACGGTGTATGTGAAGCAGCA
CCL5	F: CAGCATCAGCCTCCCCATA
	R: GGGCGGGAGAGGTAGGAAA
CXCL9	F: TGCATCAACACCAGCCAAAGGATG
	R: TTAGGCTGACCTGTTTCTCCCACT
CD19	F: CCTTCTCCAACGCTGAGTCT
	R: GGCTCAGGAAGTCAGTCGTC
ICOS	F: CGAAGACAAAGGGAAGTGGA
	R: CACTGTTATTGGATAACTGAAACTGG
CXCR6	F: GTTCTGGCCACCCAGATG
	R: GCAGACAATCATGGCAAGC
β-actin	F: AGAGCGCAAGTACTCCGTGT
	R: ACATCTGCTGGAAGGTGGAC

[1] *CCL4*: C-C motif chemokine ligand 4; *CCL5*: C-C motif chemokine ligand 5; *CD19*: CD19 molecule; *CXCL9*: C-X-C motif chemokine ligand 9; *CXCR6*: C-X-C motif chemokine receptor 6; *ICOS*: inducible T cell costimulator.

Table S3. The regulated gene ontology (GO) terms in the ileum mucosa of piglets in the amoxicillin (AM), fecal microbiota transplantation (FMT) and control (CO) groups on days 7 and 21 [1].

GO term	7 d			21 d		
	AM/CO	FMT/CO	AM/FMT	AM/CO	FMT/CO	AM/FMT
GO:0050853 | B cell receptor signaling pathway	DOWN	NO	DOWN	NO	NO	NO
GO:0030888 | regulation of B cell proliferation	DOWN	NO	DOWN	NO	NO	NO
GO:0032729 | positive regulation of interferon-gamma production	DOWN	NO	DOWN	NO	NO	NO
GO:0006955 | immune response	NO	UP	NO	UP	NO	NO
GO:0006954 | inflammatory response	NO	UP	NO	NO	NO	NO
GO:0070098 | chemokine-mediated signaling pathway	NO	UP	NO	UP	UP	NO
GO:0048247 | lymphocyte chemotaxis	NO	NO	NO	UP	NO	NO
GO:0045959 | negative regulation of complement activation, classical pathway	NO	NO	NO	NO	NO	UP
GO:0030816 | positive regulation of cAMP metabolic process	NO	NO	NO	NO	UP	NO
GO:0043950 | positive regulation of cAMP-mediated signaling	NO	NO	NO	NO	UP	NO
GO:0006691 | leukotriene metabolic process	NO	NO	NO	NO	NO	UP
GO:0009086 | methionine biosynthetic process	NO	DOWN	NO	NO	NO	NO
GO:0055085 | transmembrane transport	NO	NO	NO	NO	NO	UP
GO:0034219 | carbohydrate transmembrane transport	NO	NO	NO	NO	NO	DOWN
GO:0071918 | urea transmembrane transport	NO	NO	NO	NO	NO	DOWN
GO:0070295 | renal water absorption	NO	NO	NO	NO	NO	DOWN

[1] UP: up-regulated; DOWN: down-regulated; NO: no significant change.

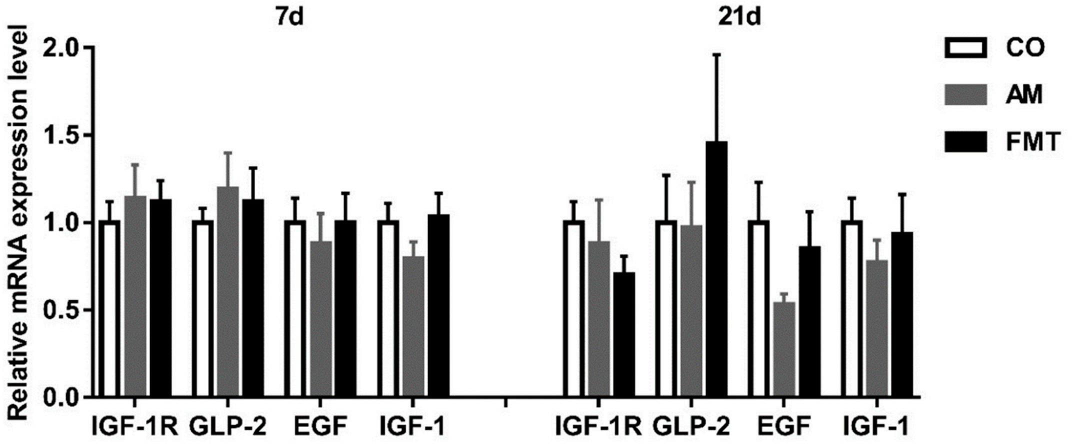

Figure S1. Effects of early intervention with maternal fecal microbiota and antibiotics on intestinal development gene of the ileum mucosa among the amoxicillin (AM), fecal microbiota transplantation (FMT) and control (CO) groups on days 7 and 21.

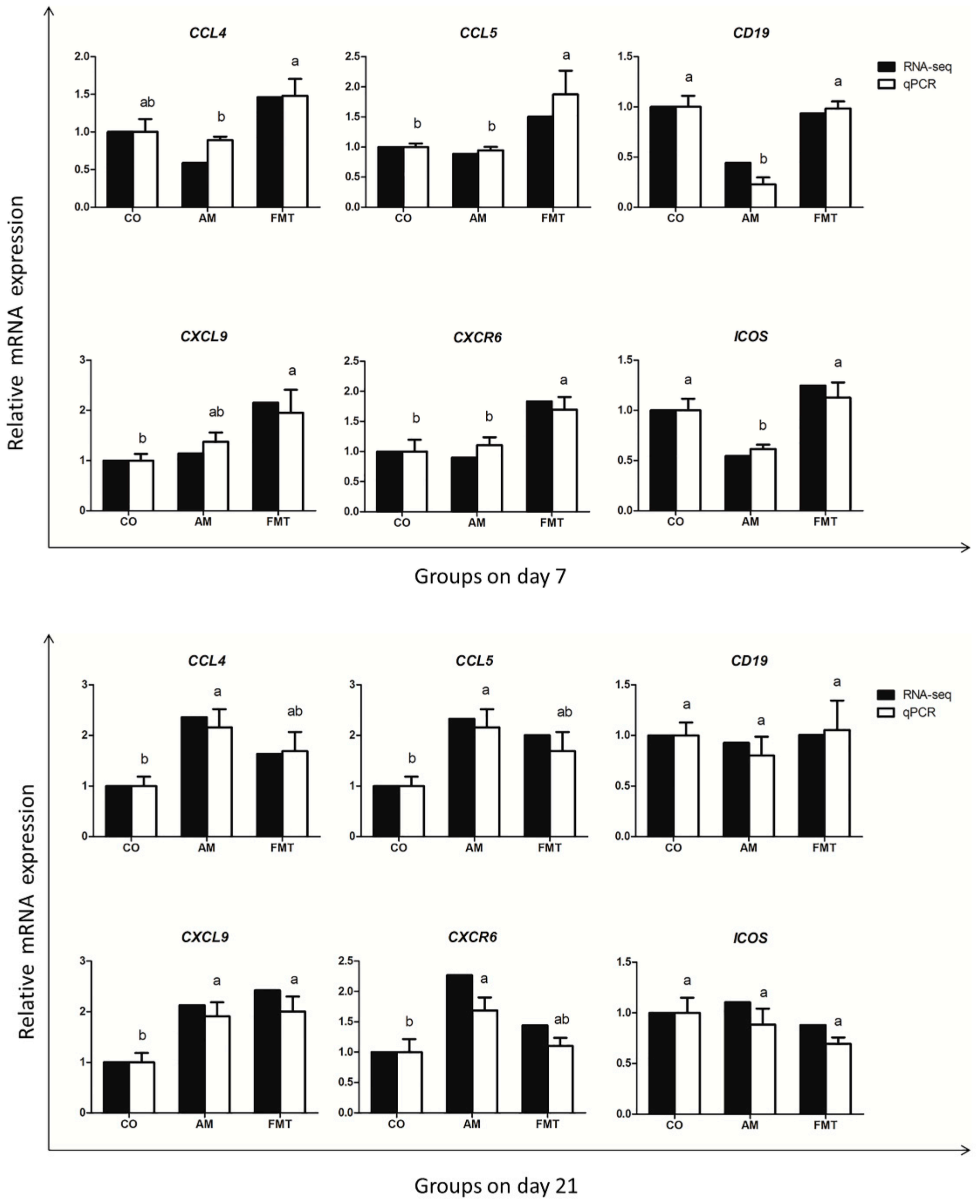

Figure S2. The qPCR validation of the RNA-seq. The results are displayed as the values of fold changes in the CO group on days 7 and 21, and the qPCR data are presented as the means ± SEM (n = 5). Values with different lowercase letter superscripts indicate a significant difference ($P < 0.05$), and those with the same letter superscripts indicate no significant difference ($P > 0.05$). CO: control; AM: amoxicillin; FMT: fecal microbiota transplantation. *CCL4*: C-C motif chemokine ligand 4; *CCL5*: C-C motif chemokine ligand 5; *CD19*: CD19 molecule; *CXCL9*: C-X-C motif chemokine ligand 9; *CXCR6*: C-X-C motif chemokine receptor 6; *ICOS*: inducible T cell costimulator.
